# Polycystic Ovary Syndrome and the Effects of a Ketogenic Diet: A Scoping Review

**DOI:** 10.3390/nu17172893

**Published:** 2025-09-07

**Authors:** Dayelise Fleigle, Jason Brumitt, Erika McCarthy, Travis Adelman, Corey Asbell

**Affiliations:** 1 Doctor of Medical Science Program, College of Medical Science, George Fox University, Newberg, OR 97132, USA; dfleigle22@georgefox.edu (D.F.); emccarthy@georgefox.edu (E.M.); tadelman@georgefox.edu (T.A.); casbell@georgefox.edu (C.A.); 2 Doctor of Physical Therapy Program, College of Allied Health, George Fox University, Newberg, OR 97132, USA

**Keywords:** hormone, KEMEPHY, ketosis, obesity, weight loss

## Abstract

**Background/Objectives**: Polycystic ovary syndrome (PCOS) is the most common endocrinopathy experienced by females. Diagnosis of PCOS is established when at least two of the following are present: hyperandrogenism, oligo-anovulation, and/or polycystic ovaries. Conservative treatment for PCOS includes dietary modifications and physical activity. The purpose of this scoping review was to evaluate the efficacy of a ketogenic diet in improving biochemical measures and reducing the severity of PCOS symptoms. **Methods**: CINAHL, PubMed, and Google Scholar databases were searched to find research published in peer-reviewed journals between 2019 and 2025. An article was included in this scoping review if the study assessed the effectiveness of a ketogenic diet on improving the signs and symptoms in patients with PCOS. **Results:** Eight studies met the inclusion criteria. Weight loss was achieved by subjects who adopted a very low-calorie ketogenic diet (VLCKD), low-calorie ketogenic diet (LCKD), classic ketogenic diet (CKD), or a Mediterranean eucaloric ketogenic diet (KEMEPHY). Patients with PCOS who consumed a ketogenic diet experienced improved biochemical measures, including androgen levels, lipid levels, HOMA-IR, blood glucose, insulin, LH/FSH ratio, DHEAS, SHBG, AFC, and AMH. A ketogenic diet was also associated with improvements in menstruation, fertility, and OHSS. **Conclusions**: Adopting a short-term ketogenic diet may have positive health benefits for patients with PCOS.

## 1. Introduction

Polycystic ovary syndrome (PCOS) is the most common endocrinopathy in females of reproductive age [1]. It is estimated that 7% of women worldwide are impacted [2]. Diagnosis of PCOS is based on the widely adopted Rotterdam Criteria [3]. Using the Rotterdam Criteria, two out of three of the following must be present to establish a diagnosis: clinical or biochemical signs of hyperandrogenism, oligo-anovulation, and/or polycystic ovaries seen on ultrasound [3]. Patients with PCOS may also present with one or more of the following: overweight/obese body mass index (BMI), infertility, insulin resistance, dyslipidemia, sleep apnea, nonalcoholic steatohepatitis, or mood disorders [1,2].

Clinical signs of hyperandrogenism include acne, hirsutism, and hair loss of the scalp [1]. Signs of biochemical hyperandrogenism are elevated serum levels of androgens, including total and free testosterone, dehydroepiandrosterone sulfate (DHEAS), and androstenedione [3]. Oligo-anovulation is defined as menstrual cycles that are >35 days apart or there are <8 menses per year [3]. On transvaginal ultrasound, ovaries are classified as polycystic if there are >12 follicles with a diameter measuring 2–9 mm in one ovary, or if the volume of an ovary is ≥10 mL [3].

Predisposing risk factors of PCOS include genetics, environmental pollutants leading to neuroendocrine dysfunction, lifestyle choices including diet, and gut dysbiosis [1]. An example of an epigenetic change includes LH/choriogonadotropin receptor hypomethylation, which leads to increased LH levels [4]. Some environmental toxins, such as endocrine-disrupting chemicals like bisphenol A, can bind to hormone receptors [4]. PCOS patients have been found to have higher levels of endocrine-disruption chemicals [4]. A combination of these risks contributes to hormonal imbalance, hyperandrogenism, and insulin resistance [1]. Neuroendocrine dysfunction affects gonadotropin-releasing hormone production by the hypothalamic–pituitary–ovarian axis, therefore unbalancing the LH:FSH ratio in PCOS patients [1]. Elevated LH contributes to hyperandrogenism, as well as theca cell hyperplasia in the ovaries, leading to polycystic ovaries [1]. Diets that are high in sugar can disrupt the gut microbiome, potentially leading to inflammation, insulin resistance, hyperandrogenism, and obesity [1].

Conservative treatment of PCOS for overweight or obese individuals involves lifestyle changes addressing diet and exercise habits [5]. Dietary weight loss of 5% has proven effective in improving metabolic factors, decreasing serum androgens, restoring ovulatory cycles, and increasing fertility in patients with obesity and PCOS [6]. There are many dietary strategies for weight loss; however, the American Academy of Family Physicians gives an evidence rating grade “A” for using a calorie-restricted diet in overweight patients with PCOS [2].

Current pharmacologic recommendations include combined oral contraceptives, antiandrogens for hirsutism, and/or metformin for insulin resistance [7]. GLP-1 receptor agonists are being increasingly utilized for weight management in PCOS patients [8]. Additionally, nutraceuticals that may offer benefit in metabolic disorders include CoQ10, vitamin D, magnesium, prebiotics, probiotics, *n*-3 polyunsaturated fatty acids, and plant sterols [9]. Inositol has also been used as an alternative to metformin in patients who were unable to tolerate gastrointestinal side effects [7,8].

The purpose of a ketogenic diet is to switch the body’s fuel source from carbohydrates to fat through induction of the metabolic state of ketosis [10]. With daily carbohydrate restriction, fasting glucose levels will decrease [10]. With limited glucose, the body breaks down fat for energy [10]. This results in the release of ketone bodies from the liver [10]. To induce ketosis, the ketogenic diet requires the consumption of low carbohydrates, moderate protein, and high fat [11]. Daily carbohydrate consumption should be limited to <50 g [12]. The classic ketogenic diet (CKD) utilizes a 4:1 ratio of grams of fat to protein and carbohydrates [13]. However, other protocols range from 1:1 to 4:1 [13]. The purpose of this scoping review is to evaluate whether a ketogenic diet is effective in improving biochemical measures and reducing the severity of the signs and symptoms of PCOS, such as irregular menstrual cycles, infertility, excessive hair growth, male pattern balding, weight gain, and insulin resistance [3].

## 2. Materials and Methods

### 2.1. Summary of Search

**Figure 1:**
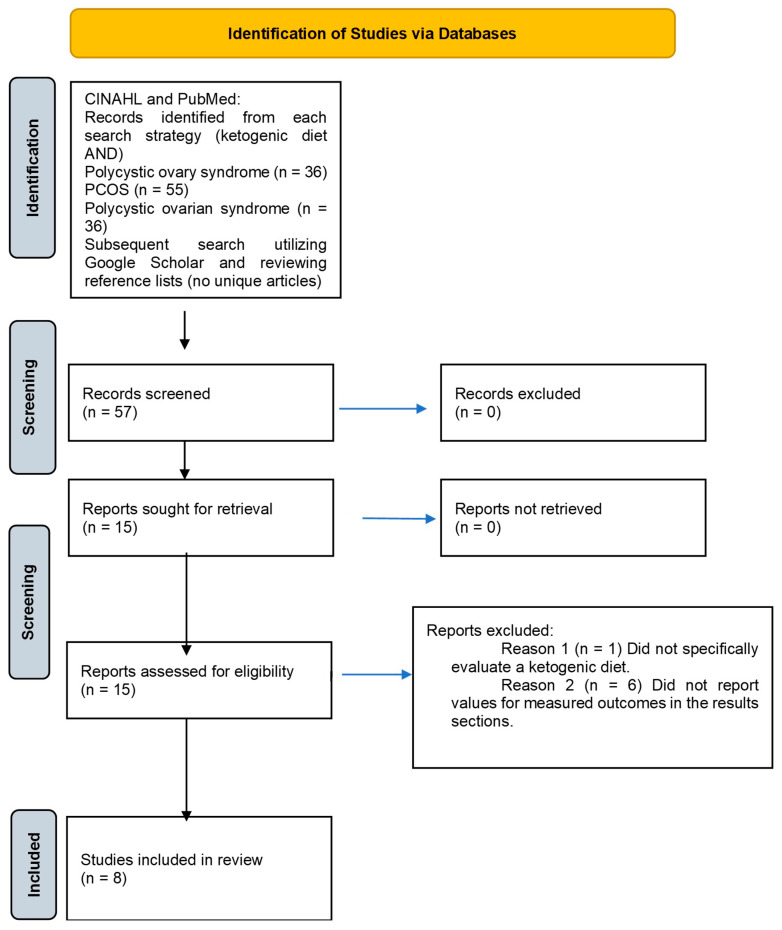
PRISMA flow diagram.

The Cumulative Index to Nursing and Allied Health Literature (CINAHL) and PubMed search engines were used to find relevant articles published in peer-reviewed journals, in English, during the prior approximate six-year period (1 January 2019–31 July 2025). The initial search of the literature (December 2024) consisted of the following keyword combinations: “ketogenic diet AND polycystic ovary syndrome,” “ketogenic diet AND PCOS,” and “ketogenic diet AND polycystic ovarian syndrome” utilizing CINAHL and PubMed (Figure 1). A subsequent search of CINAHL and PubMed using the aforementioned keyword combinations was performed in July 2025 to identify any recent publications from the initial search. In addition, a review of the references from identified articles was also performed. A secondary literature review was performed (July 2025) using Google Scholar using the same keyword combination strategies. The first 50 entries were screened for each keyword combination.

Articles were screened initially by title and abstract. An article was included in this review if it was published in English between 1 January 2019 and 31 July 2025, included patients with a diagnosis of PCOS, patients with body mass index classified as overweight or obese, and assessed the effectiveness of a ketogenic diet on improving anthropometric, biochemical, or hormonal variables. An article was excluded if it was a review article (e.g., literature review, systematic review), did not utilize the Rotterdam criteria to diagnosis PCOS, or if it did, it did not report an outcome related to anthropometric, biochemical, or hormonal variables. Two authors (DF, JB) reviewed articles for inclusion; there were no disagreements between the reviewers. A scoping review was determined to be the appropriate study design to identify relevant research. The PRISMA extension for Scoping Reviews (PRISMA-ScR) checklist was used to guide the performance of this study. This scoping review was registered with the Open Science Framework (DOI 10.17605/OSF.IO/MNHFX).

### 2.2. Results of Search

The search using the keywords “ketogenic diet AND polycystic ovary syndrome” yielded 15 results (Figure 1). After screening the articles, eight were deemed as potentially relevant. A second and third search using the keywords “ketogenic diet AND PCOS” and “ketogenic diet AND polycystic ovarian syndrome” yielded 11 and 10 results, respectively, all were repeats from the first set of identified articles. The PRISMA flow diagram summarizes the screening process (Figure 1). No unique manuscripts were identified that met the inclusion criteria after reviewing reference lists or when searching Google Scholar.

## 3. Results

Fifteen studies were assessed for eligibility. Upon full-text assessment, seven articles were excluded [7,14,15,16,17,18,19,20]. One study was excluded because it did not specifically evaluate a ketogenic diet, and six were excluded for not reporting numerical values for the measured outcomes. After exclusion criteria were applied, eight research articles remained and were included in this scoping review [7,14,15,16,17,18,19,20]. The included articles were reviewed by each author, with DF and JB extracting data included in Table 1, Table 2 and Table 3. A general overview of subjects in each study is presented in Table 1. Table 2 presents outcomes assessed and significant findings. Specific ketogenic protocols for each study are described in Table 3.

### 3.1. Weight Loss and Metabolic Parameters

Weight loss was achieved by subjects who adopted a very low-calorie ketogenic diet (VLCKD), low-calorie ketogenic diet (LCKD), CKD, or a Mediterranean eucaloric ketogenic diet (KEMEPHY) (Table 2). Meneghini et al. [14] observed a statistically significant decrease in BMI at both 90 and 120 days, Cincione et al. [17,19] at 45 days, while others recorded this finding at 8 and 12 weeks [7,15,16,18,20]. Magagnini et al. [16] reported that 76 percent of participants went from an obese BMI to an overweight BMI after following their VLCKD protocol. Patients receiving a CKD intervention [15,18,20] experienced an average weight loss ranging from -5.64 kg to −14.7 kg. Paoli et al. [7] noted a −9.43 kg decrease in body weight with the KEMEPHY, and Cincione et al. [17,19] reported a −9.5 to −11.42 kg decrease with a VLCKD intervention (Table 2). Significant improvements in body weight, BMI, and/or circumferences were observed in all studies [7,14,15,16,17,18,19,20]. Statistically significant improvements in lipid levels were observed with CKD [20], VLCKD [14,17], and KEMEPHY [7] diets. A statistically significant decrease in HOMA–IR was reported by Meneghini et al. [14] (−1.47), Paoli et al. [7] (−0.46), Cincione et al. [17,19] (−3.45, −5.70, respectively), Sharifi et al. [20] (−3.53), and Magagnini et al. [16]. Additionally, significant improvements in blood glucose were reported by Li et al. [15], Paoli et al. [7], Cincione et al. [17,19], and Sharifi et al. [20] with a variety of ketogenic protocols.

### 3.2. Hormones and Fertility

Two studies [15,18] utilizing a CKD found no change in LH and FSH; however, statistically significant increases in FSH and decreases in LH were demonstrated in studies using VLCKD [14,17,19], KEMEPHY [7], and CKD [20] protocols (Table 2). Regarding the LH/FSH ratio, Paoli et al. [7], Magagnini et al. [16], and Cincione et al. [17,19] saw significant decreases, while Meneghini et al. [14] reported it inversely as an FSH/LH ratio with a significant increase (+0.47). Statistically significant reductions in androstenedione [14] and testosterone [14,17,19] were reported with a VLCKD protocol. Reduction in serum free testosterone was noted after evaluation with VLCKD [17,19] and CKD [20] protocols. A significant decrease in DHEAS (−0.43 μg/mL) was noted with a KEMEPHY [7] ketogenic diet. SHBG was shown to significantly increase in studies by Paoli et al. [7] (+7.8 nmol/L), Magagnini et al. [16], and Cincione et al. [17,19] (+12.44 and +18.08 nmol/L). AFC was evaluated via transvaginal ultrasound in the early follicular phase, or at random for those with amenorrhea [14]. Statistically significant reductions in AFC (−5.98) and 17-alpha-hydroxy-progesterone (−0.36 ng/mL) were also seen with a VLCKD [14]. Additional results for progesterone levels were reported by multiple studies including Li et al. [15] with a significant decrease (−0.31 pmol/L), Paoli et al. [7] with a significant increase (+8.9 ng/dL), and Magagnini et al. [16] with 100% of patients having levels over 15.9 ng/mL (*p* < 0.05) on the 21st day of the menstrual cycle. Increases in estradiol were found with VLCKD [14] and KEMEPHY [7] ketogenic diets (+8.06 pg/mL, *p* > 0.05 and +52.1 pg/mL, *p* < 0.0001, respectively), while a CKD [15] protocol showed a statistically significant decrease (−15.88 pmol/L). Significant decreases in AMH were noted after 90 days [14] and 12 weeks [16], both with VLCKD protocols. The incidence rate of OHSS was reduced by 27% (*p* < 0.05), also with a VLCKD [14]. Significant improvements in the regularity of menstrual cycles among participants were recorded at 45 days [17,19], 120 days [14], and 12 weeks [15,18], with VLCKD and CKD protocols. Slight reductions in the Ferriman–Gallway scores for hirsutism were noted with a KEMEPHY [7] ketogenic diet, though it was discussed that 12 weeks may not be an adequate amount of time to see changes in the hair growth cycle.

### 3.3. Effects on Weight Loss and Metabolic Parameters

For the following analysis of data, results reported by Magagnini et al. [16] and Yang et al. [18], apart from weight loss, were not included since specific values were not retrievable. Decreases in BMI averaged a range of −1.91 kg/m^2^ to −17.03 kg/m^2^, with both extremes utilizing a VLCKD protocol [14,17] (Table 2). An average weight loss of −5.64 kg to −18 kg was observed across different diets [7,15,16,17,19,20]. Levels of total cholesterol decreased by −0.64 mmol/L with a KEMEPHY diet [7] and −40 mg/dL with a VLCKD [17], while triglycerides decreased by −7.93 mg/dL with a KEMEPHY [7] ketogenic diet to -70 mg/dL with a VLCKD [17]. HOMA-IR, measuring insulin resistance, decreased with a range of −0.46 with a KEMEPHY [7] ketogenic diet to −3.53 with a CKD [20]. Improvements of blood glucose ranged from an average of −0.46 mmol/L with a KEMEPHY [7] ketogenic diet to −0.81 mmol/L with a CKD [15]. Although it is difficult to compare results across studies, it appears that a VLCKD protocol produced the largest degree of change when analyzing these weight loss and metabolic parameters [14,16,17,19].

### 3.4. Effects on Hormones and Fertility

The LH/FSH ratio decreased from an average range of −0.47 with a VLCKD [14] to −1.99, also with a VLCKD [19] protocol (Table 2). Total testosterone ranged from a decrease of −6.06 ng/dL with a CKD [15] to −17.88 ng/dL with a VLCKD [14]. The units for total testosterone reported by Meneghini et al. [14] with a VLCKD were nmol/mL, which may have been mistaken for nmol/L, and will be interpreted as so for this review. SHBG increased from a range of 7.8 nmol/L with a KEMEPHY [7] ketogenic diet to 18.08 nmol/L with a VLCKD [17]. AMH decreased by −2.23 ng/mL with a VLCKD [14] and cannot be compared since no value was provided by Magagnini et al. [16], which was the only other study to measure it. Overall, these biochemical measures of hormones and fertility were most improved with a VLCKD protocol [14,19,20].

## 4. Discussion

### 4.1. Summary

Ketogenic diets were shown to be effective in improving biochemical measures and reducing the severity of symptoms of PCOS for patients with overweight or obese BMIs [7,14,15,16,17,18,19,20]. The improved biochemical measures of PCOS included androgen levels, lipids, HOMA-IR, blood glucose, insulin, LH/FSH ratio, DHEAS, SHBG, AFC, and AMH, while the improved symptoms of PCOS were irregular menstruation, infertility, OHSS, and weight gain [7,14,15,16,17,18,19,20]. Outcome measures that did not clearly improve across the studies were estradiol and progesterone levels [7,14,15,16]. A contributing factor to this could have been that they were measured during different phases of the menstrual cycle between studies [7,14,15,16]. A ketogenic diet is thought to be beneficial for PCOS as ketone bodies reduce appetite through the inhibition of the release of hunger hormones ghrelin and cerebral neuropeptide Y [21]. Restricting carbohydrates lowers high concentrations of insulin and serum LH [12]. The theory for LH improvement is that restricting carbohydrates improves insulin sensitivity, which affects overall endocrine function via the hypothalamic-pituitary-ovarian axis [12]. With reduced LH and improved insulin sensitivity, fewer ovarian androgens are stimulated [12]. These reductions, as well as an increase in SHBG levels, are clinically significant as they indicate an improvement of hyperandrogenism [12,14,16].

### 4.2. Ketogenic Diet vs. A Mediterranean Diet for PCOS

Conservative treatment for patients with PCOS includes dietary modifications aimed at weight loss [5]. Two potential dietary strategies are to adopt a ketogenic diet or a Mediterranean diet [11,14,19]. The Mediterranean diet has shown efficacy in achieving weight loss as well as improving metabolic and hormonal parameters [14]. Meneghini et al. [14] compared a VLCKD to a Mediterranean diet in women with PCOS. Outcome measures of weight, hormonal levels, and metabolic parameters were compared at baseline, 90 days, and 120 days [14]. The VLCKD group had greater reductions in BMI, waist-hip ratio, and abdominal circumference [14]. The VLCKD group also had lower HOMA–IR, AFC, AMH, and incidence of OHSS when compared to the Mediterranean diet group [14]. These results were achieved more quickly among the VLCKD participants [14]. Cincione et al. [19] randomized patients into a VLCKD group or a balanced hypocaloric Mediterranean diet. Both groups experienced significant improvements after 45 days in body composition and biochemical values; however, the changes in the VLCKD group were significantly greater. A VLCKD may be more beneficial to women with PCOS, especially those who are trying to conceive with time constraints [14,19].

### 4.3. Limitations of a Ketogenic Diet

The ketogenic diet can be difficult to adhere to due to intense carbohydrate restrictions [21]. If the diet is not followed, the body will no longer be in a catabolic state of ketosis [10]. Variables that may affect the outcomes of a ketogenic diet include patient comorbidities, variations among patients at baseline, types of foods that are being eaten, amount of exercise, and strictness of adherence to the diet. Side effects of a ketogenic diet are usually mild and well tolerated but may involve hypoglycemia, fatigue, headaches, nausea, vomiting, and diarrhea [21]. Meneghini et al. [14] specifically addressed if their participants experienced any side effects from the ketogenic intervention, and none reported complaints or side effects after 120 days. Contraindications for a ketogenic diet include: type 1 diabetes, eating disorders, recent stroke or heart attack, severe mood disorders, renal, hepatic, or cardiac failure [21]. Therefore, if a patient has one of these conditions in addition to PCOS, following a ketogenic diet would not be recommended. There is minimal evidence regarding adherence to a ketogenic diet long-term [21]. It is suggested that over time, patients may experience osteopenia, constipation, kidney stones, vitamin deficiencies, or dyslipidemia [11,21]. Therefore, a safe recommendation for a ketogenic diet is short-term use, around 12 weeks duration, since that is the length of time for which most research has been conducted [21].

### 4.4. Limitations of Reviewed Studies

One limitation was the variation in the timing of when progesterone and estradiol were measured during the menstrual cycle. For example, Magagnini et al. [16] measured progesterone on the 21st day of the menstrual cycle, while Li et al. [15] measured it between days three to five. This made it difficult to compare results between studies. Another limitation is the 12−week duration of the ketogenic diet protocols. While these studies identified significant short-term improvements, the long-term effects of these diets for women with PCOS are not widely known. Longer studies would provide more robust data on the long-term effects of a ketogenic diet in PCOS.

### 4.5. Future Research

Since PCOS is a chronic condition, studies evaluating the maintenance phase of a ketogenic diet, exceeding 12 weeks, could provide knowledge of the long-term outcomes patients could experience. There are limited trials regarding PCOS in adolescents. PCOS is a challenging diagnosis for adolescents as aspects of the diagnostic criteria, acne, and irregular menstrual cycles, are common [21,22]. A longitudinal study following adolescents with a diagnosis of PCOS into adulthood would be of value regarding the accuracy of diagnosis and may offer additional information that could be used to improve the diagnostic criteria. It would also be beneficial to see how early detection and treatment of PCOS in adolescents affects fertility, metabolic parameters, and hormones long-term. Additionally, future research should evaluate the impact of a ketogenic diet on the microbiome and epigenetic changes in those with PCOS [23,24,25,26].

## 5. Conclusions

Lifestyle modifications are a mainstay of treating PCOS, even more so for those wishing to take a conservative approach or who are unable to tolerate pharmacologic therapy. A ketogenic diet is one option for addressing dietary change. Even though PCOS is a chronic condition, a 12-week ketogenic diet has been shown to be beneficial for overweight or obese PCOS patients, especially with insulin resistance and while trying to conceive. The main goals of a 12-week ketogenic diet for these patients are weight loss, improved insulin sensitivity, return to menstrual cycle regularity, improved LH/FSH ratios, increased SHBG, decreased testosterone, androstenedione, and HOMA–IR. Therefore, clinicians can discuss with their overweight or obese PCOS patients the advantages and disadvantages of adopting a ketogenic diet for 12 weeks, particularly with the goal of improving biochemical measures and reducing the severity of symptoms of PCOS with non-pharmacological treatment.

## Figures and Tables

**Table 1:** Description of evaluated studies.

Author (Year) Study Design Country Conducted	Participants	Intervention
Meneghini et al. (2023) [14] Nonrandomized controlled study Country: Italy	84 patients Mean age: 33.88 y Mean BMI: 31.23 kg/m^2^	Group 1: VLCKD (42 patients) Group 2: Mediterranean diet (42 patients)
Li et al. (2021) [15] Prospective, open-label, parallel-group, randomized controlled pilot trial Country: Italy	18 patients Mean age: 27.5 y Mean BMI: 28.84 kg/m^2^	Experimental Group: classic ketogenic diet (CKD) Control Group: Conventional pharmacological treatment (Essentiale plus Yasmin)
Paoli et al. (2020) [7] Nonrandomized single-arm experimental design Country: China	14 patients Mean age: 28.90 y Mean BMI: 32.81 kg/m^2^	Mediterranean eucaloric ketogenic diet (KEMEPHY)
Magagnini et al. (2022) [16] Retrospective study Country: Italy	25 patients Mean age: 25.4 y Mean BMI: 32.8 kg/m^2^	VLCKD
Cincione et al. (2021) [17] Pre-post, single-arm study Country: Italy	17 patients Mean age: 28.5 y Mean BMI: 31.84 kg/m^2^	“Mixed ketogenic” (reflecting a VLCKD)
Yang et al. (2022) [18] Prospective cohort study, double-blind Country: China	55 patients Mean age: 27.2 y Mean BMI: 30.9 kg/m^2^	“Flexible ketogenic” (reflecting a CKD) Non-hyperuricaemia CKD group Hyperuricaemia CKD group
Cincione et al. (2023) [19] Randomized controlled trial Country: Italy	144 patients Mean age: 33.44 y Mean BMI: 33.44 kg/m^2^	Experimental group: “Mixed ketogenic” (reflecting a VLCKD) Control group: balanced hypocaloric Mediterranean Diet
Sharifi et al. (2024) [20] Randomized controlled trial Country: Iran	40 patients Mean age: 30.30 y Mean BMI: 29.21 kg/m^2^	Experimental Group: CKD Control Group: portfolio moderate carbohydrate diet

**Table 2:** Outcome measures of evaluated studies.

Author (Year)	Outcome Measures and Interval Evaluated	Anthropometric Measures	Biochemical Measures	Hormonal Measures
Meneghini et al. (2023) [14]	Evaluated at baseline and 90 and 120 days with the VLCKD: Body mass index (BMI) Waist, hip, and abdominal circumferences Luteinizing hormone (LH) Follicle-stimulating hormone (FSH) FSH/LH ratio Estradiol Androstenedione Testosterone 17-alpha-hydroxy-progesterone anti-Mullerian hormone (AMH) Antral follicle count (AFC) Risk of developing ovarian hyperstimulation syndrome (OHSS) Menstrual regularity Total cholesterol, triglycerides, high-density lipoprotein (HDL) Homeostasis model assessment for insulin resistance (HOMA–IR)	BMI decreased significantly both at 90 and 120 days: −13.16 kg/m^2^, −17.03 kg/m^2^ (*p* < 0.0001) Statistically significant reductions in waist, hip, and abdominal circumference	Decreased cholesterol and triglycerides at 90 days: −25.81 mg/dL, −17.05 mg/dL (*p* < 0.05) HDL: +4.87 mg/dL (*p* < 0.05) HOMA–IR values statistically decreased: −1.47 (*p* < 0.05)	LH: −1.40 mUI/mL (*p* > 0.05) FSH: +1.60 mUI/mL (*p* > 0.05) Increased FSH/LH ratio at 120 days: +0.47 (*p* < 0.05) Estradiol: +8.06 pg/mL (*p* > 0.05) Androstenedione and testosterone: −0.73 ng/mL, −0.62 nmol/mL (*p* < 0.05) Decreased 17-alpha-hydroxy-progesterone: −0.36 ng/mL (*p* < 0.05) Decreased AMH at 90 days: −1.79 ng/mL (*p* < 0.05) Decreased AFC: −5.98 (*p* < 0.001) OHSS risk rate was statistically lower: 27% (*p* < 0.05) 95% achieved regularity in menstruation by 120 days
Li et al. (2021) [15]	Evaluated at baseline, week 4 and 12 with CKD. BMI, body weight and composition LH FSH Progesterone Estradiol Testosterone Prolactin Total cholesterol, triglycerides, HDL, low-density lipoprotein (LDL) Glucose ALT, AST	Anthropometric Measures BMI: −4.48 kg/m^2^ (*p* < 0.05) Body weight: −11.78 kg (*p* < 0.05) Significant reductions in body fat percentage, visceral fat area, and waist-hip ratio (*p* < 0.05)	Biochemical Measures No significant differences in total cholesterol, triglycerides, HDL, and LDL levels Glucose: −0.81 mmol/L (*p* < 0.05) Significant reductions in ALT and AST (*p* < 0.05)	Hormonal Measures LH: −0.76 IU/L (*p* > 0.05) FSH: +0.44 IU/L (*p* > 0.05) LH/FSH ratio: −0.72 (*p* > 0.05) Progesterone: −0.31 pmol/L (*p* < 0.05) Significant reductions in plasma estradiol: −15.88 pmol/L (*p* < 0.05) Testosterone: −0.21 nmol/L (*p* > 0.05) Prolactin: −1.59 ng/mL (*p* > 0.05) Significant reductions in length of menstrual cycle (*p* < 0.05)
Paoli et al. (2020) [7]	Evaluated at baseline and week 12 with the KEMEPHY. BMI, body weight, and body composition Total and free testosterone LH FSH Progesterone Estradiol DHEAS Sex hormone binding globulin (SHBG) Ferriman–Gallwey score Total cholesterol, LDL, HDL, triglycerides HOMA–IR Glucose and insulin	Anthropometric Measures BMI: −3.35 (*p* < 0.0001) Body weight: −9.43 kg (*p* < 0.0001)	Biochemical Measures Total cholesterol: −0.64 mmol/L (*p* < 0.0001), LDL: −0.78 mmol/L (*p* < 0.0001), HDL: +0.23 mmol/L (*p* < 0.0001), triglycerides: −0.44 mmol/L (*p* < 0.0008) Significant improvement of HOMA–IR: −0.46 (*p* < 0.0001) Glucose and insulin: −0.46 mmol/L, −1.31 μU/mL (*p* < 0.0001)	Hormonal Measures LH: −3.83 (*p* < 0.0001) FSH: +0.45 (*p* = 0.0258) LH/FSH ratio: −0.85 (*p* < 0.0001) Progesterone: +8.9 ng/dL (*p* < 0.0001) Estradiol: +52.1 pg/mL (*p* < 0.0001) Total testosterone: −6.72 ng/dL (*p* < 0.0001) Free testosterone: −0.4 pg/mL (*p* < 0.0009) DHEAS: −0.43 μg/mL (*p* < 0.0001) SHBG: +7.8 nmol/L (*p* < 0.0001) Slightly reduced Ferriman–Gallwey scores.
Magagnini et al. (2022) [16]	Evaluated at baseline and week 12 with a VLCKD. BMI, waist circumference, and body weight Progesterone SHBG AMH HOMA–IR	Anthropometric Measures Significant reduction in BMI and waist circumference (*p* < 0.05) 76% of patients went from obese BMI to overweight BMI Mean weight loss 18 kg	Biochemical Measures HOMA–IR index values were lower than 2.5 in 96% of patient (*p* < 0.05)	Hormonal Measures Patients with progesterone levels > 15.9 ng/mL were 100% (*p* < 0.05) Significant increase in SHBG serum levels (*p* < 0.05) Serum AMH significantly decreased (*p* < 0.05)
Cincione et al. (2021) [17]	Evaluated at baseline and day 45 with a VLCKD. BMI Waist and hip circumferences with ratio Body weight LH FSH LH/FSH ratio Total and free testosterone SHBG Menstrual regularity Total cholesterol, LDL, HDL, triglycerides HOMA-IR Glucose and insulin	Anthropometric Measures BMI: −3.61 kg/m^2^ (*p* < 0.001) Waist circumference: −9.44 cm (*p* < 0.001), hip circumference: −8.10 cm (*p* < 0.001), waist hip ratio: −0.02 (*p* < 0.001) Body weight: −9.5 kg (*p* < 0.0001)	Biochemical Measures Total cholesterol: −40 mg/dL (*p* < 0.001), LDL: −35 mg/dL (*p* < 0.001), HDL: +15 mg/dL (*p* < 0.01), triglycerides: −70 mg/dL (*p* < 0.001) HOMA-IR: −3.45 (*p* < 0.001) Glucose: −10.07 mg/dL (*p* < 0.001), insulin: −12.90 μU/mL (*p* < 0.001)	Hormonal Measures LH: −4.98 mIU/mL (*p* < 0.001) FSH: +1.09 mIU/mL (*p* < 0.05) LF/FSH ratio: −1.32 (*p* < 0.01) Total testosterone: −7.34 ng/dL (*p* < 0.001), free testosterone: −0.18 ng/dL (*p* < 0.001) SHBG: +12.44 nmol/L (*p* < 0.001) Five patients with previous amenorrhea had a return of regular menstrual cycles, 12 with irregular cycles restored regularity, and five became pregnant naturally after previous failed attempts
Yang et al. (2022) [18]	Evaluated at baseline, week 6, and week 12 with CKD. BMI Body weight Body composition LH FSH LH/FSH ratio Testosterone Menstrual regularity Total cholesterol, LDL, HDL Glucose ALT, AST Uric acid	Anthropometric Measures Non-hyperuricaemia BMI: decreased (*p* < 0.001, hyperuricaemia BMI: decreased (*p* = 0.025) Non-hyperuricaemia body weight: −14.7 kg (*p* < 0.001), hyperuricaemia body weight: −11.2 kg (*p* = 0.001) Non-hyperuricaemia body fat percentage: decreased (*p* < 0.001), hyperuricaemia body fat percentage: decreased (*p* < 0.001)	Biochemical Measures Total cholesterol, LDL and HDL, had no significant differences between groups Non-hyperuricaemia glucose: decreased (*p* = 0.001), hyperuricaemia glucose: decreased (*p* = 0.013) Non-hyperuricaemia ALT: decreased (*p* = 0.001), hyperuricaemia ALT: decreased (*p* < 0.001) Non-hyperuricaemia AST: decreased (*p* = 0.007), hyperuricaemia AST: decreased (*p* = 0.005) Non-hyperuricaemia uric acid: increased significantly after 6 weeks +2.72 mg/dL (*p* < 0.001), but after 12 weeks decreased to the same basal concentration (*p* = 0.127)	Hormonal Measures LH, FSH, LH/FSH ratio, testosterone had no significant differences between groups Non-hyperuricaemia menstruation: 53.6% resumed normal menstruation, hyperuricaemia menstruation: 66.7% resumed normal menstruation
Cincione et al. (2023) [19]	Evaluated at baseline and day 45 with a VLCKD. BMI Body weight Waist circumference Total and free testosterone LH FSH LH/FSH SHBG Menstrual regularity HOMA-IR Glucose Insulin	Anthropometric Measures BMI: −4.15 kg/m^2^ (*p* < 0.001) Body weight: −11.42 kg (*p* < 0.001) Waist circumference −10.01 cm (*p* < 0.001)	Biochemical Measures HOMA-IR: −5.70 (*p* < 0.001) Glucose: −13.23 mg/dL (*p* < 0.001) Insulin: −21.64 μU/mL (*p* < 0.001)	Hormonal Measures Total testosterone: −7.40 testosterone ng/dL (*p* < 0.001), free testosterone: −0.30 ng/dL (*p* < 0.001) LH: −5.51 mUI/mL (*p* < 0.001) FSH: +2.59 mUI/mL (*p* < 0.001) LH/FSH: −1.99 (*p* < 0.002) SHBG: +18.08 nmol/L (*p* < 0.001) 25 patients with amenorrhea had a natural reappearance of a regular menstrual cycle (*p* < 0.001)
Sharifi et al. (2024) [20]	Evaluated at baseline and week 8 with CKD. BMI Body weight Waist circumference Free testosterone LH FSH DHEAS Ferriman–Gallway Score Total cholesterol, LDL, HDL, triglycerides HOMA-IR Glucose and insulin	Anthropometric Measures BMI: −2.90 kg/m^2^ (*p* < 0.05) Body weight: −5.64 kg (*p* < 0.05) Waist circumference: −5.44 cm (*p* < 0.05)	Biochemical Measures Total cholesterol −38.15 mg/dL (*p* < 0.05), LDL: −21.52 mg/dL (*p* < 0.05), HDL: +10.42 mg/dL (*p* < 0.05), triglycerides: −61.42 mg/dL (*p* < 0.05) HOMA-IR: −3.53 (*p* < 0.05) Glucose: −8.84 mg/dL (*p* < 0.05), insulin: −13.44 μU/mL (*p* < 0.05)	Hormonal Measures Free testosterone: −0.18 ng/mL (*p* < 0.001) LH: −4.38 mIU/mL (*p* < 0.05) FSH: + 0.68 mIU/mL (*p* < 0.05) DHEAS: −0.41 μg/mL (*p* < 0.05) Ferriman–Gallway Score: −0.80 (*p* = 0.12)

**Table 3:** Summary of Ketogenic Protocols per Included Study.

Author (Year)	Summary of Ketogenic Protocol
Meneghini et al. (2023) [14]	Protocol: VLCKD Intensive phase for 60 days: 800 kcal/daily Carbs: 20% (25 g) Protein: 50% (67 g) Lipids: 30% (50 g) Followed by a transition period for 30 days: 800 kcal Monday-Friday 1300 kcal Saturday 1400 kcal Sunday
Li et al. (2021) [15]	Protocol: CKD 1300–1500 calories Carbs: ≤50 g/day (5–10% of daily calories) Protein: 18–27% of daily calories Fat: 70–75% of daily calories
Paoli et al. (2020) [7]	Protocol: Mediterranean eucaloric ketogenic diet (KEMEPHY) 1672 ± 90 kcal/day 20.3 ± 5.2 g/day carbs 100.8 ± 8.6 g/day protein 132.4 ± 11.7 g/day fat 19 g per portion protein and 3.5 g per portion carbohydrate with dry phytoextracts
Magagnini et al. (2022) [16]	Protocol: VLCKD First phase: 600–800 kcal/day for four weeks Second phase: 1200–1500 for four weeks Third phase: 1500–2000 for four weeks
Cincione et al. (2021) and (2023) [17,19]	Protocol: “Mixed ketogenic” (reflecting a VLCKD) ~600 kcal daily Carbs: 30 g/day Protein: 35–40% of daily calories Fat: 30 g/day
Yang et al. (2022) [18]	Protocol: “Flexible ketogenic” (reflecting a CKD) Carbs: 5–10% of daily calories (<50 g/day) Protein: 18–27% of daily calories Fat: 70–75% of daily calories
Sharifi et al. (2024) [20]	Protocol: CKD Reduce total daily caloric intake by 500–700 k/cal Carbs: 10% of daily calories (<30 g/day) Protein: 20% of daily calories Fat: 70% of daily calories

## Abbreviations

The following abbreviations are used in this manuscript:

AMH	Anti-Mullerian hormone
ALT	Alanine aminotransferase
AST	Aspartate aminotransferase
BMI	Body mass index
CKD	Classic ketogenic diet
CINAHL	Cumulative Index to Nursing and Allied Health Literature
DHEAS	Dehydroepiandrosterone sulfate
FSH	Follicle-stimulating hormone
HDL	High-density lipoprotein
HOMA-IR	Homeostasis model assessment of insulin resistance
KEMEPHY	Mediterranean eucaloric ketogenic diet
LCKD	Low-calorie ketogenic diet
LDL	Low-density lipoprotein
LH	Luteinizing hormone
OHSS	Ovarian hyperstimulation syndrome
PCOS	Polycystic ovary syndrome
SHBG	Sex hormone-binding globulin
VLCKD	Very low-calorie ketogenic diet
